# Fats are Glossy but Does Glossiness Imply Fatness? The Influence of Packaging Glossiness on Food Perceptions

**DOI:** 10.3390/foods9010090

**Published:** 2020-01-15

**Authors:** Laura De Kerpel, Barbara Kobuszewski Volles, Anneleen Van Kerckhove

**Affiliations:** Department of Marketing, Innovation and Organisation, Faculty of Economics and Business Administration, Ghent University, 9000 Ghent, Belgium; Barbara.kobuszewskivolles@UGent.be (B.K.V.); Anneleen.VanKerckhove@UGent.be (A.V.K.)

**Keywords:** food packaging, evolved association, glossy surface, healthy inferences, consumer perception

## Abstract

This research brings together two research streams, one focusing on the influence of a diverse set of packaging attributes (e.g., shape, size, color, etc.) on perceptions of packaged food and the second one on the up- and downsides of using glossy materials, which are often studied in a non-food context. The current research deals with the influence of glossy (versus matte) food packages on consumers’ perceptions of the food inside the package. With one online survey and one quasi-experiment, we show that consumers draw inferences on the food’s fat level from the package surface, in that glossy packages are seen as a signal of fatness. This association is specific; consumers do not associate glossiness with every unhealthy product aspect. Sugar levels are unaffected by the package surface. However, due to the higher inferred fat level, a product in a glossy package is perceived to be less healthy, less tasty, and low in quality and product expensiveness. Thus, these findings suggest that glossy (versus matte) food packages mainly serve as a signal of negative product qualities.

## 1. Introduction

Imagine a consumer entering a grocery store craving for chocolate. When moving directly to the sweet aisle, the chances are this consumer spots several bars of chocolate in glossy packages and other ones with matte wrappings. Unsure about which chocolate bar to pick from the assortment, this consumer is drawn to one in a matte package, assuming that the glossy one was of the fatty, low-quality type. The present research is occupied with verifying whether this person’s inferences are widely applied by consumers. That is, this research investigates whether package surface glossiness influences: (1) consumers’ beliefs on the packaged product’s fat content and (2) derived beliefs on the product’s healthiness, quality, expensiveness, and taste.

By studying the product beliefs that result from glossy product packages, we contribute to the body of literature on the communicative potential of package elements. Existing research has, for example, addressed the influence of packaging materials on product beliefs [1,2]. That is, different packaging materials, like paper versus plastic wrappings, have been shown to give rise to diverse product expectations in terms of expected quality, taste, and healthiness [3]. In a similar vein, this research expands this body of work by focusing on an under-researched package element, namely the glossiness of package surfaces.

There are only a few studies on glossy (versus matte) package coatings. Research by Ye, Morrin, and Kampfer [4] provides initial evidence for package glossiness to serve as an informative cue to the fat content of the product inside. The authors suggest that learned associations are driving the inferences on fat content that are drawn from glossiness. More specifically, they argue that over time consumers have learned that greasy foods are sold in glossy packages. The current research provides a more comprehensive understanding of the link between package surface and product inferences by studying beliefs on both the fat level and the sugar level of products, considering that in the marketplace not only fatty products but also sugary products are sold in those glossy packages. Hence, current consumer environments also foster a ‘glossy = sugary’ association, though consumers do not seem to endorse this association.

By adopting this more comprehensive approach, we not only shed another light on the scope of inferences from glossy packages, but we also ascribe the link between glossiness and fat to an evolved rather than a learned association. If the effect were indeed caused by a learned association, then package glossiness would not only increase expectations on the product’s fat level but also those on its sugar level should increase. However, we find that package glossiness leaves sugar content perceptions unaffected, which is in line with an evolutionary psychology account. That is, when an evolved association (i.e., our ancestors learned to attend to glossiness as it is an outward sign of a resource important for survival, namely fat) is underlying the effect, the effect should only manifest in terms of fat content beliefs.

### 1.1. Food Package Design

Food package design can serve as a medium to communicate the properties of the product inside the package [2,5]. While grocery shopping, consumers often derive product expectations from a product’s visual appearance [5,6]. These expectations can pertain to low-level attributes, like the product’s constituents, or to higher-level, overarching expectations related to product quality, for example [7]. Several packaging elements, like color (i.e., hue, saturation, luminance) [8,9], shape [10], material [11], and surface [12] have been addressed in extant research [13]. For example, watered-down or “lighter” colored packages signal that the product inside is healthier; packages with more vibrant colors lead the packaged product to be seen as more attractive [14]. Note, however, that product impressions are often informed by a myriad of sensory experiences. A growing body of literature shows that the taste of food, for example, is derived from multiple sensory cues, including smell, vision, sound, and touch, rather than just taste [15]. Similarly, a product’s glossiness is not only inferred from its visual appearance, but also from haptic cues. A product that feels slippery is judged to be glossier [16]. Hence, the cues provided by each of the senses tend to be combined to constitute the perceptions of a product’s attributes [17]. Overall, this body of work suggests that when consumers are exposed to certain package elements, this elicits expectations on the product inside [1,18].

A diverse set of product beliefs, ranging from perceptions pertaining to product health and taste to product quality expectations, has been shown to be, at least in part, derived from product package design elements. For example, package shape (i.e., angular versus rounded packages, anthropomorphizing a package shape to mirror an ideal human body shape) and package color (i.e., less saturated versus more saturated colors) drive taste beliefs and even actual taste experiences [7,19,20]. Package colors can also influence the product’s perceived healthiness, as research established highly saturated colors (versus lowly saturated colors) to improve health perceptions [21]. Moreover, package size influences quality beliefs in that smaller (versus larger) packages are thought to be of higher quality [22]. Based on this, studying the inferences consumers draw from other packaging elements, like packaging surface, is a topic worthy of further research.

### 1.2. Glossy Versus Matte Packaging Materials

Glossy objects catch the eye of human beings [23]. In everyday life, two different reasons may be underlying these positive responses toward glossiness. On the one hand, people are keen on the use of gloss as it is aesthetically appealing. Indeed, Meert, Pandelaere, and Patrick [24] document a preference for glossy pictures over matte ones. To explain this attraction to glossy, they rely on an evolutionary viewpoint stating that this preference for glossy stems from a human need for fresh water necessary to survive [24]. On the other hand, a glossy surface might also serve as an informative cue. For example, Fleming, et al. [25] show that gloss perception enables people to estimate 3D shapes of objects. The visual perception of glossy packages leads to inferences on haptic impressions. While glossy packages are thought of as thinner and lighter, matte ones are expected to feel rougher [26]. These haptic perceptions can then, in turn, lead products in glossy packages to be seen as more sophisticated [26] and higher in quality [27]. Likewise, glossy packages are deemed to signal a product’s luxury position [28]. Specifically, with respect to food products (i.e., fish), Murakoshi et al. [29] identified that humans infer the freshness of food from glossiness (i.e., the luminance distribution in an image) as glossiness is an indicator of the wetness of the surface of the food object.

While this body of research seems to suggest that package glossiness leads to positive inferences in a non-food context (and serves as a freshness indicator for highly perishable food), recent research states that for rather artificial food products, matte (instead of glossy) packaging is preferred as the matte coating affects the perceived naturalness of the product that is inside the package [12]. Similarly, research by Ye et al. [4] establishes an association between glossy packages and the products’ perceived healthiness. Additionally, their findings extend existing research by providing evidence of the underlying process [4]. According to these authors [4], learned associations have created expectations regarding the healthiness of packaged foods. More specifically, through repeated exposure in the marketplace, consumers have noticed and consequently learned that unhealthy or greasy products are sold most often in glossy packages, whereas matte packages mostly contain healthier products [4]. However, in the current marketplace, not only greasy products but also products with a high level of sugar are wrapped in those glossy packages. As such, when a learned association is indeed sufficient to cause the effect, we would expect the glossy coating of the package to increase consumers’ expectations on both the perceived level of greasiness and the perceived level of sugar.

In this study, however, we argue that a learned association is not sufficient to explain this effect. Extant (neurological) research found that for primate species information from a product’s surface appearance, such as surface glossiness, can provide cues for the perception of the material and quality of the food [30,31]. In line with this finding, we believe that a glossy package serves as a cue to evaluate the product that is inside. While previous research in a non-food context links the preference for glossy to an innate need for water [24], we hypothesize that, in a food context, a glossy surface may also remind consumers of grease. In other words, we believe that the positive association between package glossiness and greasiness of the product might exist because fat and glossiness share some exterior resemblance. Consequently, when the association is evolved rather than a learned one, no effect of a glossy package on the perceived level of sugar is expected as sugar and glossiness do not share these external characteristics.

### 1.3. Research Aims and Hypotheses

In two studies, we investigate how packaging glossiness can influence food product evaluations. As fat has a glossy outlook, our ancestors realized they had to attend to glossiness to help them survive. Considering this exterior resemblance between fat and glossiness, we propose that wrapping a product in a glossy package might increase expectations on the product’s fat level compared to a matte package. Prior research, however, attributes this effect to a learned association between fat and glossiness as this combination can be frequently found in food packaging [4]. As sugary products are often sold in glossy packages as well, a learned association would also imply an increase in a product’s expected sugar level when wrapped in a glossy package. In this paper, though, we do not expect an effect of packaging glossiness on sugar-level inferences as sugar and glossiness do not share an exterior resemblance. As such, we believe that an evolved, rather than a learned association is underlying the effect.

**Hypothesis** **1.** **(H1)** Glossy (versus matte) food packaging increases expectations on the packaged product’s fat level, but not on the packaged product’s sugar level.

It has been established that a positive package label (e.g., organic or low in calorie) enables consumers to form a favorable product attitude and ultimately favorable inferences about other, unknown attributes of the product [32]. The opposite can also occur. Research on a negative halo or devil effect demonstrates that a negative label (e.g., artificial ingredients) causes higher calorie estimates, which then leads to negative health inferences [33]. Similarly, if consumers indeed link a glossy package to a high fat content, we hypothesize that this then leads to inferences of low product quality (and expensiveness). In line with previous research, we will also assess how packaging glossiness impacts credence attributes, which, even after consumption, cannot be readily observed, such as product naturalness [12] and healthiness [4]. Furthermore, we investigate the effect of package glossiness on the actual taste experience. By examining different packaging elements, consumers are also able to form certain expectations about the taste of food products [34,35,36], which then affect actual consumer experiences and judgments [37,38]. As such, we hypothesize that:

**Hypothesis** **2.** **(H2)** Glossy versus matte food packaging decreases product evaluations and health inferences. More specifically, we expect glossy (versus matte) food packaging to increase a product’s fat-level inferences and decrease:

(a) Expected product quality
(b) Expected product expensiveness
(c) Expected product healthiness
(d) Actual taste experience

Given that prior research on packaging glossiness has addressed perceived naturalness of the product [12], we also take this measure into account. We hypothesize that consumers attribute high fat levels to products wrapped in glossy packages. As fat is a natural ingredient, products in glossy packages might accordingly be seen as more natural than those with low fat levels. Extant research, however, found matte rather than glossy packages to signal naturalness, at least for artificial products [12]. As such, we do not formulate any a priori expectations on a product’s perceived naturalness when it is wrapped in glossy compared to a matte package.

To test the hypotheses, two studies were carried out. First, a survey was conducted to verify whether consumers indeed associate glossy packages with both fatty and sugary snacks. Second, a quasi-experiment was set up to verify the effect of packaging glossiness on expected product evaluations and health inferences. The experiment also evaluated actual taste experiences. 

## 2. Study 1: Survey

To gauge which types of snacks consumers relate to glossy packages, we gathered survey data. Specifically, in line with extant research [4], we expect to find that consumers associate snacks with higher fat levels with glossier packages. However, we argue that consumers have learned many more associations with glossy packaging. For example, high sugary snacks are also often sold in glossy packages. Hence, we propose that consumers have above average expectations of both types of snacks to be packaged in glossy (versus matte) packages.

### 2.1. Method

Data for this study were collected via Amazon’s Mechanical Turk platform (*N* = 184, 77 men, *M*_age_ = 39.92, *SD* = 12.99). First, two types of products that differ in terms of fat and sugar content were identified. That is, we are interested in packaging associations for products with a (1) high sugar, but low-fat content, and products with a (2) low sugar, but high fat content. We identified sweets as products exemplar of the first product type whereas potato chips are considered exemplar of the second product type. Then, we adapted the statements of Ye, Morrin, and Kampfer [4] to measure the extent to which participants associate these product types more with glossy packaging than with matte packaging. Participants responded to three seven-point semantic differential items: “Potato chip [sweets] packages usually have a matte surface–glossy surface,” “I have come to expect potato chips [sweets] to be sold in matte packaging–glossy packaging,” and “Most potato chips [sweets] are sold in matte packs–shiny packs” (Cronbach’s α_potato_chips_ = 0.90, Cronbach’s α_sweets_ = 0.94). In the survey, the order in which participants answered questions was counterbalanced as such that half of the respondents first indicated its responses for the potato chips, followed by the sweets, whereas the other half of the participants received the questions in the opposite order.

### 2.2. Results and Discussion

Agreement with the statements was averaged across the three items, per product type. As predicted, participants believed that potato chip packages have an above average tendency to have a glossy surface (*M* = 5.70, *SD* = 1.32, one-sample *t* (183) = 17.45, *p* < 0.001). The same thing, however, goes for sweets packages. Even though this product type has rather low-fat levels, participants do believe that sweets are predominantly sold in glossy packages (*M* = 5.49, *SD* = 1.44, one-sample *t* (183) = 14.02, *p* < 0.001). Moreover, there is no difference between packages’ glossiness expectations of potato chips and sweets (*M*_potato_chips_ = 5.70, *M*_sweets_ = 5.49, paired-samples *t* (183) = 1.52, *p* = 0.13). In this study we find that both fatty and sugary products are associated with glossy packages. If a learned association is sufficient for package glossiness to induce product inferences, then glossy packages should drive inferences on products’ fat and sugar levels. However, if the effect is due to an evolved association between fat and glossiness, then we should find that the effect is specific for fat level inferences and does not generalize to sugar level inferences, despite the learned associations identified in this study. In the next study, the product-level characteristics that consumers infer from package glossiness are addressed.

## 3. Study 2

The second study was set up to uncover whether glossy packages indeed trigger inferences on the fat level of the product inside the package, without triggering inferences on the sugar level. To this end, participants are exposed to either matte-packaged or glossy-packaged chocolates in a store. The product was positioned on a separate table and participants were given the opportunity to inspect the product package visually and haptically. As research has shown that product perceptions are influenced by multiple sensory inputs, it seemed appropriate to allow participants to touch the product packages, as touch likely has an important influence; it might be even more important than vision [17].

### 3.1. Method

This quasi-experimental research was set in a branch of a large European retailer. Shoppers frequenting this branch aged 18 or more were invited to participate when passing by the sampling counter. Over a three-day period, 178 shoppers (72 men; *M*_age_ = 52.40, *SD* = 15.63) participated in the study.

**Figure 1:**
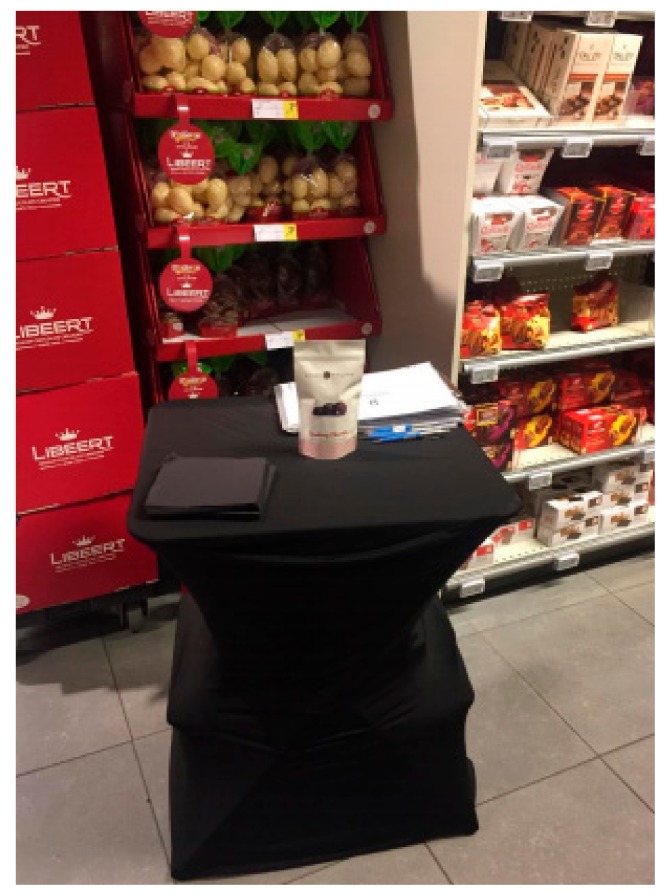
Sampling booth in front of a chocolate assortment in store, displaying one package prototype.

**Figure 2:**
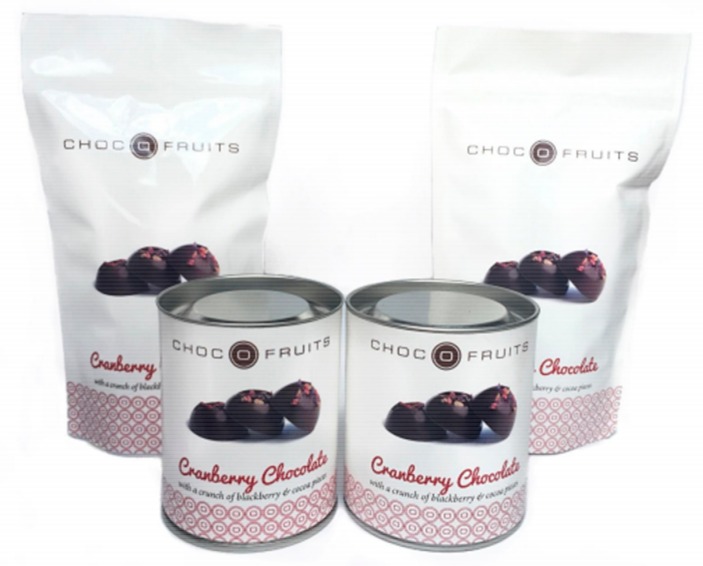
Stimulus material used in study 1: glossy packaging (left), matte packaging (right).

On the sampling counter, one package of chocolates was presented (see Figure 1). The chocolates (and the packages) were prototypes of a new type of chocolates that was not yet available on the market. Hence, none of the customers could have been familiar with this product, nor with its packaging. The pack of chocolates appeared in one of four possible forms. Two packaging elements varied across conditions. The first factor, which is the focal element in this study, pertains to the package surface that was either glossy or matte. The second factor relates to the shape of the package; this study features two package shapes, namely bags and cylinders. This second factor was added to the design to avoid finding idiosyncratic effects suggesting that only glossy bags, for example, and no other glossy packages instigate fat level inferences (see Figure 2 for a picture of the packages that were used in this study), as prior research indicated that inferences derived from visual cues might differ according to package shape [20]. However, we had no a priori expectations on, nor explicit interest in, the effect of package shape on consumers’ product inferences. As the study was set in-store, it was not possible to randomly assign shoppers to one of four conditions. Instead, a quasi-experimental design was implemented where shoppers were assigned to a condition based on the time of the day at which they were shopping for groceries. In order to avoid confounds due to different types of shoppers frequenting a store at different moments, we switched the focal package every hour and the packages were displayed on different moments over three days. That is, if a package had been displayed during the first time slot on day 1, it would be displayed during the second time slot on day 2, for example. An overview of the presentation scheme can be found in Table 1.

Standing at the sampling booth, shoppers were first given the opportunity to inspect the package, visually and haptically. Next, they were asked to complete questions on their expectations of the chocolates inside. Most importantly, participants reported on a seven-point semantic differential scale their expectations on the product’s fat level by responding to the item “In comparison to other chocolates, I assume that the chocolates inside the presented package contain a lot of fat–little fat”. In addition, because we formulated specific expectations on inferences about the fat content, we also measured expectations on the product’s sugar content. If glossiness indeed signals fattiness in particular, we should find no difference in expectations on the product’s sugar level. Specifically, participants indicated to what extent they assumed that “In comparison to other chocolates, the chocolates inside the presented package contain a lot of sugar–little sugar”. Participants also reported their perceptions of the overall healthiness, quality, expensiveness, and naturalness of the product, all on one-item seven-point semantic differential scales. Measures of overall perceived healthiness, quality, and expensiveness were included to verify whether changing expectations on the product’s fat level also influences higher-level evaluations of the product. Naturalness perceptions were measured because Marckhgott and Kamleitner [12] found glossy packages to reduce perceived naturalness of products. After reporting perceptions, participants were given the opportunity to sample the chocolates and to report taste experiences on a two-item (“tastes bad–tastes good,” “unappetizing–appetizing,” Pearson’s *r* = 0.93) seven-point semantic differential scale [39].

### 3.2. Results and Discussion

Even though the study was not built according to a typical repeated measures design, a repeated measures Analysis of Variance (ANOVA) was used to test the first hypothesis. And all data were analyzed using IBM SPSS Statistics 25 (IBM Corp., Armonk, NY, USA). ANOVA statistical test allowed us to gauge the interaction between a between-subjects (package surface: glossy versus matte) and a within-subjects variable (health inference: fat content and sugar content). Specifically, a repeated measures ANOVA, estimating the main effects and interaction effect of the factors package surface (glossy versus matte) and package type (bag versus cylinder) on fat-level and sugar-level beliefs, was run. In line with our expectation that package surface would influence expectations on the product’s fat-level, but not on the product’s sugar-level, the occurrence of an interaction effect between this between-subjects factor and the within-subjects factor is verified and confirmed (*F*(1,174) = 22.31, *p* < 0.001). Specifically, when considering fat-level expectations, we found these to be higher for glossy (*M* = 3.89, *SD* = 0.92) versus matte packages (*M* = 3.21, *SD* = 0.97, *F*(1,174) = 23.32, *p* < 0.001). A glossy (*M* = 5.92, *SD* = 1.14) versus matte (*M* = 6.22, *SD* = 0.82) package surface also seemed to instigate a (smaller) difference in the anticipated sugar-level, albeit in the opposite direction (*F*(1,174) = 4.45, *p* = 0.036). Other than a main difference between fat-level and sugar-level expectations, no significant within-subjects effects could be observed (all *p’s* > 0.05).

A multivariate two-way ANOVA, to verify the effect on higher-level inferences, was run. Via this analysis, the main effects and interaction effect of package surface (glossy versus matte) and package type (bag versus cylinder) on overall healthiness, quality and expensiveness, and the average reported taste experience was estimated. As formulated in hypothesis 2, we anticipated all of these variables to be lower for glossy versus matte packages, whereas we did not have specific anticipations on the effect of package type. In fact, the latter factor was included in this study to avoid reporting idiosyncratic effects, and, as such, our main interest resided in the presence or absence of interaction effects between package type and package surface, but not so much in the effect of package type itself. The multivariate test results point to a significant main effect of package surface (*F*(4,171) = 12.09, *p* < 0.001) and package type (*F*(4,171) = 4.50, *p* = 0.002), but no significant interaction effect (*F*(4,171) = 1.44, *p* = 0.223). We further gauged the main effect of package surface by interpreting the univariate test results that are presented in Table 2. Overall, a matte package tends to result in higher expectations on the product’s health, expensiveness, and quality and even improves actual taste experiences. Without being the focal element of this study, the main effects of package type are also relevant to mention. They are appended to Table 2. It appears that the cylindrical packaging yields more positive product inferences, only the observed difference in terms of anticipated product quality ratings is not significant.

Interestingly, even though the finding that a matte package yields more positive product perceptions than a glossy package (cf. supra) is in line with the finding of Marckhgott and Kamleitner [12], we did not find evidence that this was due to a difference in perceptions of product naturalness in this study. A two-way ANOVA with perceived naturalness as the dependent variable and package surface and package type as the predictors returns non-significant main and interaction effects (all *p’s* > 0.05). Most importantly, the perceived naturalness of the product in the matte package (*M* = 3.03, *SD* = 0.88) was not significantly different from the perceived naturalness of the product in the glossy package (*M* = 2.97, *SD* = 1.11, *F* (1,174) = 0.08, *p* = 0.782). It has been argued that a difference would only be observed for products of which the baseline naturalness perceptions is not high [12]. It is difficult to determine in hindsight whether these rather low ratings of perceived naturalness can still be considered as too high to observe a difference. However, we suggest that this might be one reason why we did not observe a difference on this aspect in this study. The results of this study are, however, a first step in providing evidence for an alternative route via which perceptions of products in matte packages can be improved, namely via reducing inferences on products’ fat level.

## 4. General Discussion

### 4.1. Summary of Findings

The current research demonstrates that a glossy compared to a matte package leads to higher expectations of the product’s fat content, which, in turn, leads to lower ratings of the product’s healthiness, expensiveness, and quality. A glossy package even deteriorates actual taste experiences. Furthermore, we find evidence that the pattern of observed findings is more in line with an evolved rather than a learned association between glossiness and fat content. More specifically, Study 1 shows that in everyday life a positive association exists between glossy packages and the perceived fat level of the product inside the package. Moreover, Study 1 shows that consumers also associate glossy packages with sugary products. As such, based upon the theory of learned associations, one would expect that packaging glossiness influences consumers’ expectations on both the product’s fat and sugar level. The results of Study 2, however, demonstrate that consumers make fat level inferences, but not sugar level inferences from packaging glossiness, which is in line with an evolutionary perspective.

### 4.2. Theoretical and Practical Contributions

Prior research has shown that many different packaging elements serve as extrinsic cues that are informative of the products inside. To date, literature on the effectiveness of packaging elements has focused mostly on elements such as color [9,40], size [41,42], shape [19,43], and their combined influence on the consumer’s multisensory product experience [7,17,44]. Packaging surface, in contrast, has received only limited attention. Some studies have investigated the use of glossiness, though this research was primarily set in a non-food context [24,28]. Only scant research has actually combined these two streams by investigating the influence of glossy packages on the food product that is wrapped inside. As such, this paper extends food packaging literature by showing that the glossy (versus matte) coating of a package might elicit different consumer responses. More specifically, the results show that a glossy (versus matte) package signals a lower quality and expensiveness of the food product wrapped inside. This finding is in contrast with research on non-food objects, which suggests that a glossy surface of such an object is positively related to luxury and consequently conveys an image of greater quality [28]. In a non-food context, this preference for glossiness is attributed to an innate need for water [24]. In a (perishable) food context, research by Murakoshi et al. [29], confirmed the positive effect of glossiness by demonstrating that glossiness signals freshness of the food as the glossy surface is an indicator of water content. As this paper, however, deals with less perishable food, the freshness of the food might be less of a concern. Rather, we argue that, in a non-perishable food context, glossiness might also remind consumers of fat. Due to these different underlying mechanisms, opposite findings can be expected when comparing the use of glossiness in both contexts. Furthermore, we confirm previous research [4], as we show that glossy packages indeed signal lower perceived healthiness of the product. In contrast, we cannot confirm the Marckhgott and Kamleitner [12] findings stating that consumers attribute a higher perceived naturalness to products wrapped in a matte (versus glossy) package. Additionally, our findings contribute to the use of evolutionary psychology frameworks in consumer research [19,45,46]. Research by Ye et al. [4] documents that consumers learned to associate glossy packages with products containing high fat levels through repeated exposure. In other words, the authors believe that a learned association between fat and glossiness is explaining the effect [4]. The results of Study 1, though, show that nowadays consumers expect not only fatty, but also sugary products to be wrapped in glossy packages. Thus, if a learned association is indeed underlying the effects, then glossy packages should drive inferences on both products’ fat and sugar levels. In Study 2, however, we find that the effect is specific for fat level inferences and does not generalize to sugar level inferences. As such, we propose that an evolved, rather than a learned, association is a key element from driving the negative effects between packaging glossiness and product evaluations. By no means do the reported findings discard the role that a learned association may play. We argue that a learned association is not a sufficient explanation for all that we observe; it might play a complementary role, though. For example, what once originated from an evolutionary association may persist or become more pronounced because of the frequent association of two concepts (gloss and fat) in our current surroundings. Moreover, while previous research in a non-food context has shown that humans’ preference for glossiness might stem from an innate need for fresh water [24], we propose an alternative explanation. Indeed, one could argue that, glossy surfaces in a food context might also remind consumers of grease or fat. By documenting this evolved association between fat and glossiness, we extend previous research on evolutionary psychology.

As such, our findings create relevant guidance for public policy makers who aim to stimulate healthy food consumption among consumers. Because of the increasing obesity rates among adults and children [47], upgrading the image of healthy food in the minds of consumers is crucial. Therefore, our findings suggest that healthy food can be promoted in shopping contexts by enhancing the taste beliefs of healthy food by using matte packages. This may stimulate consumers’ perception of healthy foods and help food manufacturers that produce healthy foods to flourish in the market.

### 4.3. Limitations and Future Research

This research raises a couple of interesting issues that could be addressed in the future. A first issue involves the products that are used. In Study 1, we limited our research to the use of two unhealthy snacks, namely sweets and potato chips. In this way, we could confirm that consumers expect both sugary (i.e., sweets) and fatty (i.e., potato chips) products to be sold in glossy (versus matte) packages. Nevertheless, it remains to be tested whether our results are generalizable to different sugary and fatty products. Moreover, further research might be needed to check whether packaging glossiness also influences evaluations of natural products. Prior research by Marckhgott and Kamleitner [12] has shown that packaging glossiness only diminishes products’ perceived naturalness when they are not perceived as natural already. In Study 2, though, we could not even observe a difference in perceived naturalness for a rather artificial product (i.e., chocolates). We concluded that the product’s low ratings of perceived naturalness might still be too high to observe a difference. Lastly, future research could make a difference between saturated (i.e., bad) and unsaturated (i.e., good) fats. While at first sight fat content is a less salient property for healthy products, these foods might still be high in unsaturated fats (e.g., avocados). As such, the question remains whether packaging glossiness elicits different reactions when the product inside is generally believed to be high in unsaturated compared to saturated fats.

Second, we implemented a quasi-experimental design in Study 2. This study was set in-store and shoppers were assigned to a condition based upon the time of the day at which they were grocery shopping. As no random assignment takes place, quasi-experimental research cannot eliminate the problem of confounding variables. To avoid any confounds due to different types of shoppers frequenting a store at different moments, the focal package was switched every hour and the packages were displayed on different moments over three days. Although we tried to account for possible confounds, the use of a true experiment might still be more desirable when considering internal validity.

Third, we find evidence that a learned association is not sufficient to explain consumers’ negative evaluations of products wrapped in glossy (versus matte) packages. As such, we contradict prior research by Ye et al. [4]. As a product’s expected fat level but not sugar level increases when wrapped in a glossy package, we suggest that it is more likely that an evolved association between fat and glossiness is driving the effects. However, this research lacks direct evidence to prove the existence of such an evolved association. Consequently, it might be worthwhile for future research to weigh these two different explanations against each other.

Fourth, the question rises whether consumer traits, such as health consciousness, moderate the effect of packaging glossiness on product evaluations. Health-conscious consumers actively monitor and adjust their state of health [48]. To achieve their overarching health-goal, health-conscious consumers are more sensitive to extrinsic cues indicating health benefits [49,50]. As such, we argue that the negative effect of glossy packages on product evaluations should be strengthened for health-conscious consumers as they pay more attention to such an extrinsic cue. This proposition is in line with research by Van Loo et al. [51], which states that consumers with a greater interest in sustainability visually attend more to sustainability information on food packages.

Fifth, further research might be needed to check whether packaging glossiness can indeed be seen as attention-grabbing. Han [52] found that glossy packages are more successful in grabbing consumers’ immediate attention compared to matte packages. However, the question remains whether glossy packages are still equally successful in grabbing attention when they are grouped together on the same display. Besides the attention-grabbing potential, it might also be worthwhile to take into account other behavioral measures. With the exception of actual taste experiences, we only tested the influence of packaging glossiness on consumer expectations toward the product. Therefore, it might be an interesting avenue for future research to investigate whether glossy packages also have an impact on actual (e.g., buying or consumption) behavior.

Finally, our findings do not completely align with previous research stating that objects with glossy surfaces are perceived as more luxurious [28]. Our research shows that the use of glossiness in a product packaging context may backfire. Indeed, we document that glossy packages trigger inferences on the fat level of the product inside the package, which then negatively influences consumers’ expectations on the product’s price and quality. In general, low-priced products signal lower product quality [53]. Perceived product quality, however, might be conditional upon the interplay between the glossiness of the package and the price asked for the product. More specifically, we believe that glossy wrapping for low-priced food products might lower consumers’ quality expectations even more. In a similar vein, wrapping high-priced food products in glossy packaging could potentially raise perceptions of quality even further.

Addressing aforementioned issues in further research might help us to get more insight in the impact of packaging glossiness on product evaluations.

## Figures and Tables

**Table 1:** Package presentation scheme.

	9 h	10 h	11 h	12 h	13 h	14 h	15 h	16 h	17 h	18 h	19 h
Day 1	1	2	3	4		1	2	3	4	1	2
Day 2	3	4	1	2		3	4	1	2	3	4
Day 3	2	3	4	1		2	3	4	1		

1 = glossy cylinder, 2 = matte cylinder, 3 = glossy bag, 4 = matte bag.

**Table 2:** The effect of package surface and package type on higher-level evaluations: Descriptions and test results.

**Independent Variable: Package Surface**						
	Glossy		Matte		*F*	*pP*
	*M*	*SD*	*M*	*SD*		
Health	4.08	0.97	4.74	0.81	24.22	<0.001
Quality	5.09	0.96	5.99	0.79	46.18	<0.001
Expensive	4.82	1.11	5.61	0.89	27.11	<0.001
Taste	5.07	1.17	5.92	0.83	31.78	<0.001
**Independent Variable: Package Type**						
	Cylinder		Bag			
	*M*	*SD*	*M*	*SD*	*F*	*pP*
Health	4.60	0.79	4.22	1.05	8.13	0.005
Quality	5.62	0.94	5.44	1.03	1.72	0.192
Expensive	5.42	0.98	5.01	1.14	7.29	0.008
Taste	5.75	0.86	5.24	1.24	11.32	0.001
